# Metabolomic study of Chilean biomining bacteria *Acidithiobacillus ferrooxidans* strain Wenelen and *Acidithiobacillus thiooxidans* strain Licanantay

**DOI:** 10.1007/s11306-012-0443-3

**Published:** 2012-07-21

**Authors:** Patricio Martínez, Sebastián Gálvez, Norimasa Ohtsuka, Marko Budinich, María Paz Cortés, Cristián Serpell, Kenji Nakahigashi, Akiyoshi Hirayama, Masaru Tomita, Tomoyoshi Soga, Servet Martínez, Alejandro Maass, Pilar Parada

**Affiliations:** 1 BioSigma S.A., Loteo Los Libertadores, Lote 106, Colina, Chile; 2 Laboratory of Bioinformatics and Mathematics of the Genome, Center for Mathematical Modeling (UMI 2807, CNRS) and Center for Genome Regulation, University of Chile, Avda. Blanco Encalada 2120, 7th Floor, Santiago, Chile; 3 Institute for Advanced Biosciences, Keio University, Tsuruoka, Yamagata Japan; 4 Department of Mathematical Engineering and Center for Mathematical Modeling (UMI 2807, CNRS), Faculty of Mathematical and Physical Sciences, University of Chile, Avda. Blanco Encalada 2120, 7th Floor, Santiago, Chile

**Keywords:** Bioleaching, Metabolomics, Biomarker, Capillary electrophoresis, Mass spectrometry, CE–MS, *Acidithiobacillus*, *Thiooxidans*, *Ferrooxidans*, Wenelen, Licanantay, Spermidine

## Abstract

In this study, we present the first metabolic profiles for two bioleaching bacteria using capillary electrophoresis coupled with mass spectrometry. The bacteria, *Acidithiobacillus ferrooxidans* strain Wenelen (DSM 16786) and *Acidithiobacillus thiooxidans* strain Licanantay (DSM 17318), were sampled at different growth phases and on different substrates: the former was grown with iron and sulfur, and the latter with sulfur and chalcopyrite. Metabolic profiles were scored from planktonic and sessile states. Spermidine was detected in intra- and extracellular samples for both strains, suggesting it has an important role in biofilm formation in the presence of solid substrate. The canonical pathway for spermidine synthesis seems absent as its upstream precursor, putrescine, was not present in samples. Glutathione, a catalytic activator of elemental sulfur, was identified as one of the most abundant metabolites in the intracellular space in *A. thiooxidans* strain Licanantay, confirming its participation in the sulfur oxidation pathway. Amino acid profiles varied according to the growth conditions and bioleaching species. Glutamic and aspartic acid were highly abundant in intra- and extracellular extracts. Both are constituents of the extracellular matrix, and have a probable role in cell detoxification. This novel metabolomic information validates previous knowledge from in silico metabolic reconstructions based on genomic sequences, and reveals important biomining functions such as biofilm formation, energy management and stress responses.

**Electronic supplementary material:**

The online version of this article (doi:10.1007/s11306-012-0443-3) contains supplementary material, which is available to authorized users.

## Introduction

Several extremophiles have been isolated from mining operations (Johnson et al. 2001; Okibe et al. 2003; Tyson et al. 2005), and their role in the dynamics and evolution of minerals has previously been discussed (Santelli et al. 2009). They are known to have a relevant role in hydrometallurgic extraction processes: their presence is linked to enhanced extraction of metals such as copper, nickel, cobalt, zinc and uranium. This process, termed “biomining” or “bioleaching”, is an example of industrial biotechnology as an empirical process, governed by trial testing. In 1947, the phenomenon of metal dissolution in acid media was attributed to microorganismal action (Colmer and Hinkle 1947). Biomining is described as the extraction of metal from sulfide ores or concentrates by the action of acidophilic bioleaching microorganisms that catalyze mineral ore oxidation. These microorganisms are naturally present in the minerals’ native flora. The industrial process is designed to provide an environment with optimal growth conditions in order to optimize microorganismal action. Dump and heap bioleaching operations using this technology are located in SBL Radomiro Tomic in Chile, Cerro Verde in Perú, Morenci in USA, among others.

The use of specific microorganisms and/or their derivatives is relatively new to this industry. It has been shown that these enhance conventional processes of acid irrigation several fold, which eventually translates into economical benefits for companies (BioSigma US Patent No. 7,601,530; 7,700,343; 7,837,760 among others). Studies of their physiology are crucial for understanding how they interact with the mineral surface and how they can be optimized to improve mineral dissolution.

Acidophilic prokaryotes used for metal recovery from sulfide minerals include members of the Bacteria and Archaea domains. We have isolated several microorganisms with the aim of using them in biomining processes for copper extraction. The two selected isolates show improved oxidizing activity when compared to standard international strains: these are *A. ferrooxidans*, strain Wenelen DSM 16786, and *A. thiooxidans*, strain Licanantay DSM 17318 (Sugio et al. 2009; Ohata et al. 2010). The former is an iron and sulfur oxidizing microorganism, while the latter is strictly sulfur oxidizing. Detailed studies of both chemolithotrophic bacteria have been undertaken since their isolation in 2003 (Levicán et al. 2008), and continue to be a matter of interest in other “omics” analyses.

An important complication when studying biomining microorganisms is genetic transformation. Notwithstanding, there are some reports of successful transformations, which are considered random phenomena (Liu et al. 2000; Kusano et al. 1992). This explains why, despite advances made in past years, little is known regarding the specifics of their metabolism. Genomic sequences analyzed with bioinformatics tools have provided insights into their metabolism (Valdés et al. 2008; Cárdenas et al. 2010; Quatrini et al. 2009). The information gathered from these sequencing projects complement other “omics” data, such as gene expression microarray experiments, mass spectrometry based metabolite detection and proteomics (Ishii et al. 2007). The latest advances in metabolomics, particularly the quantitative metabolic response, are attributable to high-throughput techniques, which separate and detect cellular compounds. One such technique, named capillary electrophoresis Time-of-Flight Mass Spectrometry, CE–TOFMS, has several advantages including: high resolution, quantification of charged low molecular weight compounds, and suitability for different organisms (Soga et al. 2003, 2006; Sato et al. 2004). However, there are limitations related to metabolomic coverage (Ohashi et al. 2008).

In this paper, we report the first metabolomic study of bioleaching microorganisms. Different mineral substrates were tested as energy sources for both bioleaching isolates, *A. ferrooxidans* strain Wenelen and *A. thiooxidans* strain Licanantay. The aim of the study is to reveal information about the metabolic pathways of these two bioleaching bacteria. In addition, we compare their growth in ideal conditions (pure media energy sources—iron and sulfur) to their growth under more realistic conditions (chalcopyrite and ore impurities). Finally, we compare cells attached to solid substrate versus free ones, as results could reveal information on contact and non-contact bioleaching.

High-throughput data analysis highlighted differences between the metabolic profiles of the bacteria when faced with different energy sources. Similar conclusions are drawn when comparing different cell populations. Standard metabolite analysis reveals that specific metabolites are abundant and can be secreted to the extracellular space.

## Materials and methods

### Strains and growth conditions

Two isolates obtained from mining environments, *A. ferrooxidans*, strain Wenelen (DMS 16786), and *A. thiooxidans*, strain Licanantay (DMS 17318), were used in this study (Sugio et al. 2009; Ohata et al. 2010).

*Acidithiobacillus ferrooxidans* strain Wenelen, an iron/sulfur oxidizing bacteria, was grown in KDM media containing (NH_4_)_2_SO_4_ 0.99 g/l, NaH_2_PO_4_ *2H_2_O 0.145 g/l, MgSO_4_ *7H_2_O 0.10 g/l, KCl 0.10 g/l, CaCL_2_ 0.021 g/l, KH_2_PO_4_ 0.052 g/l with either FeSO_4_ 6 g/l, 1 % sulfur or 1 % concentrate (composed mainly of chalcopyrite, CuFeS_2)_ obtained from a Chilean mine. For sulfur oxidizing *A. thiooxidans* strain Licanantay, KDM was supplemented either 1 % sulfur or 1 % concentrate from a Chilean mine. The mineral was sterilized 3 times by autoclave at 120 °C for 30 min. Both strains were cultivated in bioreactors at 30 °C with a pH of 1.8 under all conditions. Liquid cultures were stirred at 150 rpm with an aeration flow of 0.5 VVM (volume per volume per minute).

### Metabolite extraction protocol

Two reactors were managed under the same conditions for each microorganism in order to obtain biological replicates.

Samples were taken at three time points (T1, T2 and T3) corresponding to the exponential, early stationary, and late stationary phase, respectively (Supplementary Fig. SF1).

Our protocol is a modified version of the Soga et al. (2002) protocol.

For solid substrate growing conditions (sulfur and chalcopyrite), 200 ml of the culture were filtered using a vacuum pump with 2 filters in tandem: the upper filter had a 5 μm pore size to retain cells attached to the substrate (sessile cells), and the lower filter (0.2 μm pore size) retained free cells (planktonic cells). For soluble substrate (iron) only the lower filter was used.

To clean samples, we performed two washes with 10 ml of acidic water (pH 1.8), followed by two additional washes with distilled water.

Filters were immersed in a methanol solution (5 ml) with three internal standards: methionine sulfone, 2-(*N*-morpholino) ethane sulfonic acid (MES) and d-camphor-10-sulfonic acid (CSA).

Cells were sonicated for 30 s and then incubated for 10 min in order to quench enzymatic reactions. 4 ml of the homogenate were mixed with 1.6 ml water and 4 ml chloroform, and then vortexed and centrifuged for 5 min at 3,222 RCF at 4 °C. The aqueous phase, containing intracellular metabolites, was collected and subjected to centrifugal filters with a 5 kDa cut-off (millipore).

Sample solutions were centrifuged at 9,520 RCF at 4 °C for 2 or 3 h. Next, the filtrate was evaporated by the centrivac system until samples were dry.

Samples were stored at −80 °C until CE–MS analysis.

For supernatant analysis, samples were collected from the reactor at the same time points (T1, T2 and T3). The two filtering steps consisted of: a 0.2 μm millipore filter to remove cells, and then, a second filtration using a centrifugal filter (5 kDa). Samples were dried and stored at −80 °C until CE–MS analysis.

Controls for all assays were generated following the same protocol without cells.

### Analytic conditions for metabolome analysis

The setup conditions for all runs were performed as described by Hirayama et al. (2009) with some modifications. Three technical replicates were carried out for each sample.

### Instruments

All CE–TOFMS experiments were performed using a CE capillary electrophoresis system equipped with TOFMS, 1100 isocratic HPLC pump, G1603A CE–MS adapter kit, and G1607A CE–electrospray ionization (ESI)–MS sprayer kit (Agilent Technologies). System control and data acquisition were performed using Agilent G2201AA ChemStation software and Analyst QS for CE and TOFMS, respectively. In addition, the original Agilent SST316Ti stainless steel (Fe/Cr/Ni/Mo/Ti; 68:18:11:2:1) ESI needle was replaced with a platinum needle to avoid poor robustness and needle corrosion (Soga et al. 2009).

### CE–TOFMS conditions for cationic metabolite analysis

Cationic metabolites were separated with a fused-silica capillary (50 μm i.d. 100 cm total length) filled with 1 mol/L formic acid as the reference electrolyte. Sample solution was injected at 50 mbar for 3 s (ca. 3 nL), at 30 kV. The capillary and sample trays were maintained at 20 °C and below 5 °C, respectively. Sheath liquid was composed of methanol/water (50 % v/v) with 0.1 μmol/L hexakis (2,2-difluorothoxy)phosphazene (Hexakis) delivered at a rate of 10 μL/min. ESI–TOFMS was operated in the positive ion mode. Capillary voltage was set at 4 kV and the nitrogen gas flow rate at 10 psig (heater temperature 300 °C). In TOFMS, the fragmentor, skimmer, and octapole radio frequency voltage (Oct RFV) were set at 75, 50, and 125 V, respectively. An automatic recalibration function was performed according to the mass of two reference standards: ^13^C isotopic ion of protonated methanol dimer ([2CH_3_OH +H]^+^, *m*/*z* 66.06371) and protonated Hexakis ([M + H]^+^, *m*/*z* 622.02896), which provided the lock mass for exact mass measurements (acquired at a rate of 1.5 cycles/s over a 50 to 1,000 *m*/*z* range).

### CE–TOFMS conditions for anionic metabolite analysis

Anionic metabolites were separated using a cationic-polymer-coated SMILE(+) capillary (Nacalai Tesque) with 50 mmol/L ammonium acetate (pH 8.5) as the reference electrolyte. Sample solution was injected at 50 mbar for 30 s (ca. 30 nL) at −30 kV. Ammonium acetate (5 mmol/L) diluted in 50 % methanol/water (50 % v/v) containing 0.1 μmol/L Hexakis, was used as sheath liquid at 10 μL/min. ESI–TOFMS was operated using the negative ion mode. The capillary voltage was set at 3.5 kV. In TOFMS, the fragmentor voltage, skimmer voltage, and Oct RFV were set at 100, 50, and 200 V, respectively. An automatic recalibration function was performed according to the mass of two reference standards: ^13^C isotopic ion of deprotonated acetate dimer ([2CH_3_COOH-H]^−^, *m*/*z* 120.03841) and Hexakis + deprotonated acetate ([M + CH_3_COOH-H]^−^, *m*/*z* 680.03554). Other conditions were identical to those used in cationic assay.

### Standard metabolites

A mix of 112 metabolites (Supplementary Table ST1), with known *m*/*z* and migration times, was used as a standard for sample identification and quantification. All standards were of analytical grade and obtained from Wako, Aldrich or Sigma.

### Data processing

Preliminary raw data analysis for experimental conditions was performed with the MasterHands Program (Sugimoto et al. 2010a, b). Once conditions were adjusted, data in wiff format were converted to mzXML and exported to the MeltDB platform (Neuweger et al. 2008). The platform was adapted for CE–MS dataset storage, as it was originally designed for GC/MS and LC–MS data management. Peak detection was performed using the XCMS software (Smith et al. 2006). Peak detection parameters, “signal to noise ratio” threshold and peak “full width at half maximum”, were optimized for the anionic and cationic samples. Global normalizations were performed using spiked internal standards for standard runs and biological samples (Ishii et al. 2007). Since migration time variations are significant in capillary electrophoresis (Soga et al. 2006), we aligned chromatograms along the time axis in order to compare CE–MS runs. We adjusted retention times for each chromatogram by an ad-hoc methodology. First, we located internal sample standards, selecting the largest peak area for matching *m*/*z*. Then, a sample retention time correction was performed by linear adjustment using internal standards as reference. Next, we searched for compatible *m*/*z* and retention times for the remaining 112 standards; if a standard fit, it was regarded as present. A quadratic model was used to re-correct retention times using all present standards. Finally, we performed the last round of standard detection and a final quadratic correction for retention times using all localized standards (Supplementary Table ST2).

We excluded peaks found in control samples in order to remove peaks lacking a biological origin.

Relative peak areas detected in standard runs were used to derive metabolite concentrations in biological sample runs. Intracellular concentration was calculated using the estimated weight of a cell (2.80 × 10^−13^ g), its volume (4.96 × 10^−16^ l) and cell concentration (Ishii et al. 2007). For estimation purposes, all cells were considered free cells.

### Multivariate data analysis

Principal component analysis of the complete dataset was performed using the R software. These analyses considered detected peaks as variables and their abundance as values. Zero abundance values were assigned to conditions where peaks were not detected. Data was centered and scaled before these analyses.

## Results and discussion

The ultimate goal of this study is to expand knowledge on key active metabolic pathways in *A. ferrooxidans* strain Wenelen and *A. thiooxidans* strain Licanantay. We compared their growth under ideal conditions (pure media and energy sources—iron or sulfur) to more realistic conditions and energy sources (chalcopyrite and ore impurities). We also contrasted free and attached cells, as this comparison could highlight differences between contact and non-contact bioleaching.

The data analysis is divided into two sections: first, we evaluate the complete dataset, including peaks representing known and unknown metabolites, and then we outline discoveries based on metabolites identified for each condition.

### Complete dataset analysis

Tables 1 and 2 summarize the complete dataset after peak detection and control peak subtraction for *A. ferrooxidans* strain Wenelen and *A. thiooxidans* strain Licanantay samples, grown on their respective energy sources. In general, fewer peaks were detected in attached cell samples compared to free cell samples for both bacteria. The presence of more mineral residues in attached cell samples may interfere with the ionization process. The area of the detected peaks was smaller for free cell samples for *A. thiooxidans* strain Licanantay grown in chalcopyrite, in both anionic and cationic modes. This behavior, however, was not observed for samples grown in sulfur.

**Table 1:** Average number of peaks and their normalized areas for *A. thiooxidans* strain Licanantay sample runs grown on either chalcopyrite (Cpy) or elemental sulfur (S^0^)

Energy source	Sample type	Anionic mode	Cationic mode
Cpy	Free cells		
	Average number of peaks	142 ± 28	58 ± 104
	Average normalized area [×10^3^]	1.99 ± 0.54	1.84 ± 0.41
	Attached cells		
	Average number of peaks	133 ± 12	442 ± 53
	Average normalized area [×10^3^]	1.24 ± 0.24	3.88 ± 1.03
S^0^	Free cells		
	Average number of peaks	161 ± 65	520 ± 214
	Average normalized area [×10^3^]	4.54 ± 3.07	3.04 ± 1.55
	Attached cells		
	Average number of peaks	73 ± 25	360 ± 42
	Average normalized area [×10^3^]	2.27 ± 1.11	1.97 ± 0.42

**Table 2:** Average number of peaks and their normalized areas for *A. ferrooxidans* strain Wenelen sample runs grown on either Fe or elemental sulfur (S^0^)

Energy source	Sample type	Anionic mode	Cationic mode
Fe^+2^	Free cells		
	Average number of peaks	143 ± 32	423 ± 38
	Average normalized area [×10^3^]	4.92 ± 4.66	2.31 ± 0.19
S^0^	Free cells		
	Average number of peaks	126 ± 8	244 ± 71
	Average normalized area [×10^3^]	1.17 ± 0.27	2.34 ± 1.39
	Attached cells		
	Average number of peaks	126 ± 8	192 ± 49
	Average normalized area [×10^3^]	1.01 ± 0.24	1.83 ± 1.09

Principal component analysis (PCA) was performed using the complete dataset to determine if the metabolomic data allowed for separation of the different conditions. Principal components were calculated using all detected peaks (for each sample) as variables and their abundance as values.

#### Cationic CE–MS runs

**Fig. 1:**
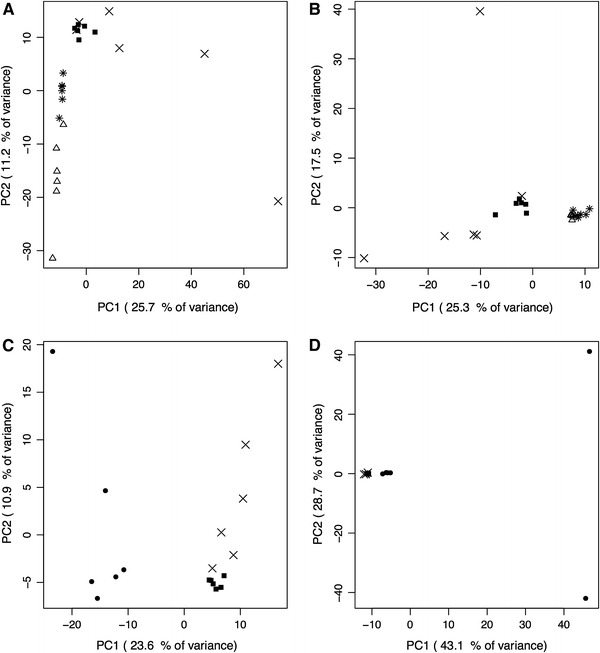
PCA for *A. ferrooxidans* strain Wenelen and *A. thiooxidans* strain Licanantay samples grown on either iron, elemental sulfur or chalcopyrite energy sources. **a, c** *Data* from cationic runs. **b, d** *Data* from anionic runs. **a, b** *A. thiooxidans* strain Licanantay sample analysis. **b, d** *A. ferrooxidans* strain Wenelen sample analysis. *Multiplication symbol* sulfur attached cells, *filled square* sulfur free cells, *asterisk* chalcopyrite attached cells, *open triangle* chalcopyrite free cells, and *filled circle* iron free cells

Figure 1c shows clear separation between sulfur and iron conditions for *A. ferrooxidans* strain Wenelen samples. Separation observed in sulfur samples indicates that the two filters used in the extraction protocol allow cell populations (free and attached cells) to be distinguished. Similarly, positive values in PC1 are strongly associated to unique peaks detected in sulfur samples. This shows that unique features are present under both conditions and can be used as possible biomarkers for each condition. For *A. thiooxidans* strain Licanantay sample separation was observed for different energy sources (Fig. 1a). Attached and free cell samples were separated in chalcopyrite growing conditions, but not in sulfur.

#### Anionic CE–MS runs

For *A. ferrooxidans* strain Wenelen anionic runs, PCA failed to clearly separate the various growth conditions (Fig. 1d). This is due to the presence of two outlying chromatograms originating from Fe cultures. These chromatograms are associated to cultures in late exponential growth, where on average, the number of detected peaks is more than three times the average for all other samples. This phenomenon suggests an increased adduct abundance.

*Acidithithiobacillus thiooxidans* strain Licanantay samples grown in sulfur and chalcopyrite conditions can be differentiated as shown in Fig. 1b. However, as with cationic runs, attached and free cell samples were not separated.

Differences in samples obtained during different growth stages were not clearly reflected by PCA of CE–MS runs in either mode.

### Annotated standard metabolites analysis

**Fig. 2:**
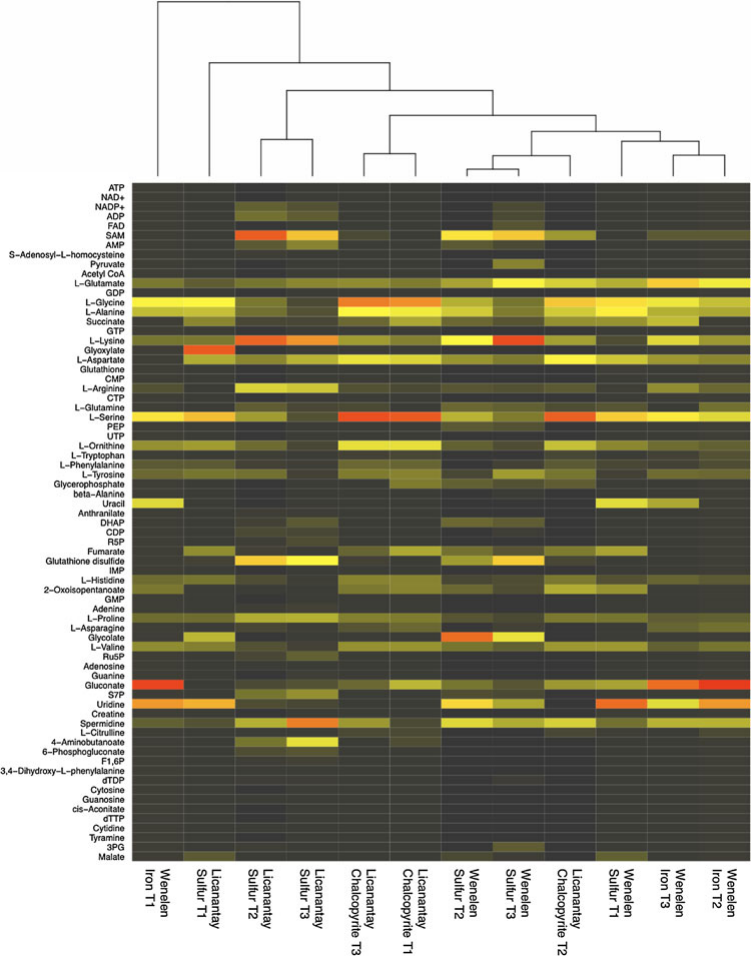
Heatmap of metabolites detected in each experiment. *Each matrix column* has been rescaled: *black* is the *lowest value*, *yellow* represents the *middle value* and *red* is the *maximum value* for intracellular concentration. W, *A. ferrooxidans* strain Wenelen; L, *A. thiooxidans* strain Licanantay (Color figure online)

In cationic and anionic chromatograms, a search for a set of 112 standard metabolites (Supplementary Table ST1) was made and those detected were annotated. The intracellular concentrations of these annotated metabolites in free cells were calculated (Fig. 2).

As expected, sulfur and iron conditions in *A. ferrooxidans* strain Wenelen are grouped in T2 and T3, respectively, exhibiting differing metabolic profiles according to growth media. Samples in T1 show different behavior, most likely because fewer metabolites are detected as a result of low cell concentration (Fig. 2). Also, *A. thiooxidans* strain Licanantay in chalcopyrite shares characteristics with *A. ferrooxidans* strain Wenelen in sulfur conditions, suggesting similarities in sulfur processing.

Spermidine appears ubiquitously across different conditions. Glutathione disulfide, which is of particular importance for sulfur assimilation (Rohwerder and Sand 2003), is present in both organisms under sulfur growth. Gluconate characterizes ferrous growth of *A. ferrooxidans* strain Wenelen, and moreover, glutamate, tryptophan and phenylalanine show particular profiles depending on the growth media.

#### Metabolic pathways

Metabolic pathways were reconstructed and characterized. We paid special attention to the polyamine synthesis pathway because it seems active in every energy condition: we analyzed the presence of spermidine in the supernatant, which could have a significant role in this pathway (Supplementary Table ST3).

Metabolites present in glutathione pathways, which are related to elemental sulfur oxidation, were also analyzed given their activity in sulfur conditions.

Glutamate, aspartate and various metabolites involved in energy processes were detected in supernatants. Then, we analyzed the amino acid profiles for each microorganism.

The list of metabolites detected and their intracellular concentrations is provided in the supplementary material (Supplementary Table ST4). In addition, a summary of metabolic pathways containing these metabolites is included (Supplementary Figs. SF2, SF3; Supplementary Table ST5).

#### Polyamines

Polyamines are policationic compounds present in all cells, specifically found in the intracellular space (Tabor and Tabor 1985; Cohen 1998). These metabolites are involved in a variety of biological responses such as cellular proliferation, differentiation, biofilm formation (Igarashi and Kashiwagi 1999), protein synthesis (Friedman and Oshima 1989), and DNA synthesis and stabilization (Terui et al. 2005).

In our study, levels of *S*-adenosyl-l-methionine (SAM), a spermidine synthesis intermediate, are below the detection threshold in early growth stages (T1 in Table ST4). However, levels are detectable in exponential and stationary phases under all conditions (T2 and T3 in Table ST4). Spermidine, which is also present in bacterial supernatants, is detected 5 to 6 fold with respect to SAM in the corresponding growth phases, indicating a tendency for accumulation. Interestingly, intracellular spermidine levels in *A. ferrooxidans* strain Wenelen are approximately one-third higher in iron compared to sulfur media (Table ST4). Due to the soluble state of iron, spermidine accumulates in the intracellular space because of insufficient secretion. In contrast, sulfur media offers solid surface stimuli, which allows extracellular spermidine secretion, and therefore, less intracellular accumulation. For *A. thiooxidans* strain Licanantay, we expected that both solid energy sources (sulfur and chalcopyrite, Table ST4) would enhance biofilm production and have similar spermidine secretion and concentration tendencies. However, secretion was only observed in sulfur conditions (Table ST3).

Arginine and ornithine, two other intermediates described in the spermidine biosynthesis pathway, show opposing behaviors for most cases: arginine production increases, whereas ornithine decreases over time (Table ST4).

**Fig. 3:**
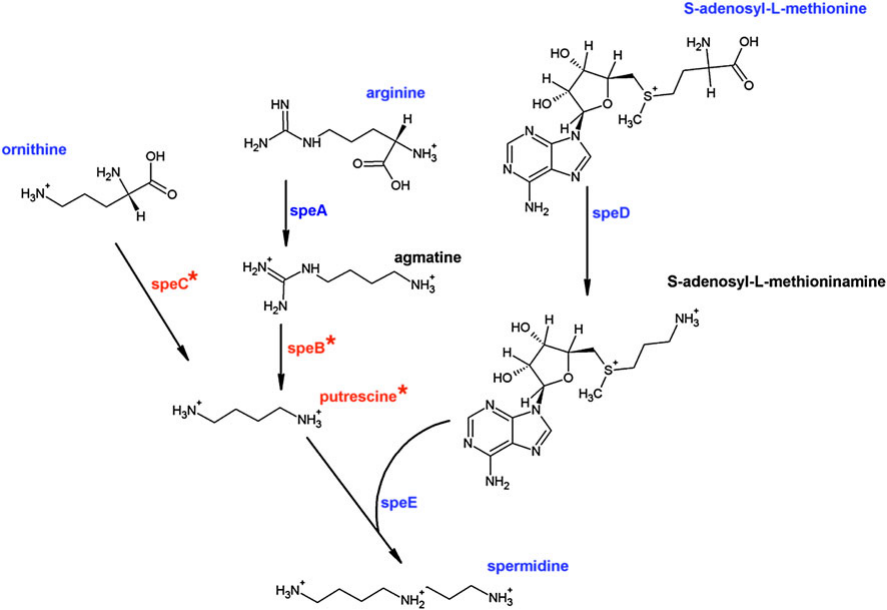
Spermidine synthesis pathway. Spermidine is produced from putrescine and *S*-adenosyl-l-methioninamine. Ornithine and arginine are precursors of putrescine. *speA* arginine decarboxylase; *speB* agmatine ureohydrolase; *speC* ornithine decarboxylade; *speD* *S*-adenosyl-methionine decarboxylase; *speE* spermidine synthase. *Blue* detected compounds and genes in *A. ferrooxidans* strain Wenelen and *A. thiooxidans* strain Licanantay; Red* undetected compounds and genes (Color figure online)

Two common polyamine synthesis routes have been described in bacteria (Tabor and Tabor 1985), citing ornithine and arginine amino acids as precursors (Fig. 3).

Most mesophilic microorganisms produce the most common polyamines: putrescine, spermidine and spermine (Tabor and Tabor 1985; Cohen 1998). Nevertheless, a variety of new polyamines and synthesis routes have been described mainly in thermophiles from Archaea and Bacteria domains (Ohnuma et al. 2005; Terui et al. 2005).

Our metabolic studies have produced data that indicate an alternative route for polyamine synthesis. The common bacterial spermidine intermediate, putrescine, was not detected in any condition (detection limit for putrescine: 0.1 uM), which suggests that spermidine synthesis might be analogous to routes described in other extremophiles (Ohnuma et al. 2005).

For both bacteria under study, we searched for genes related to polyamine synthesis (Fig. 3). *speA*, arginine decarboxylase, *speD*, SAM decarboxylase and *speE*, polyamine agmatine aminopropyl transferase or spermidine synthase, were found in the microorganismal genomes. Interestingly, *speB* (agmatine ureohydrolase) was not detected, which reinforces the absence of putrescine in the metabolic data. *speC* (ornithine decarboxylase) was also absent, which suggests that there could be an alternative pathway for spermidine synthesis which has not yet been reported in these bacteria.

To determine *spe* gene expression levels under various growth conditions, we analyzed data collected in *A. ferrooxidans* strain Wenelen microarray experiments (data not shown). Results show slightly higher *spe* gene expression in sulfur media: gene expression is higher for *speE* and *speD* in sulfur and chalcopyrite compared to tetrathionate (S_4_O_6_^2−^) and iron conditions. *speA* does not show expression changes for any condition, which suggests it is a constitutive gene. The presence of spermidine in the supernatant (sulfur condition, Supplementary Table ST3) leads us to believe that it could be acting as a communication molecule for bacterial adhesion on insoluble energy sources (through biofilm formation). Biofilm synthesis and polyamines have been described in bacterial genera such as *Yersinia*, *Streptococcus*, and *Vibrio* (Haugo and Watnick 2002; Shah et al. 2006). Polyamines could have an important role in cell-to-cell signaling in *Proteus mirabilis* (Sturgill and Rather 2004) and *Vibrio cholerae* (Karatan et al. 2005). This is consistent with spermidine abundance in *A. ferrooxidans* strain Wenelen and *A. thiooxidans* strain Licanantay supernatants in sulfur conditions. Spermidine, however, was not detected in *A. thiooxidans* strain Licanantay supernatant under the chalcopyrite condition (Table ST3). This could be due to low amounts of bioavailable sulfur on the mineral surface, which cannot induce spermidine secretion or attachment. Biofilm formation in *A. ferrooxidans* is closely related to the production of extracellular polysaccharides (Sand and Gehrke 1999), and its composition changes according to the energy substrate (Gehrke et al. 1998). The genes proposed to be involved in this pathway (*gal* operon) show differential expression when bacteria are growing in either sulfur or iron (Barreto et al. 2005). The changes in extracellular polysaccharide composition seem to be related to attachment mechanisms, which are used by microorganisms in different energy sources. At this level, spermidine may be regulating sulfur attachment and biofilm formation mechanisms. Also, spermidine found in supernatant could be linked to a detoxification response: the toxic effect of intracellular spermidine accumulation has been studied in *Escherichia coli* (Fukuchi et al. 1995). Moreover, a new excretion protein complex (Higashi et al. 2008), which is functional and specific for spermidine detoxification, has recently been reported.

The consistent presence of spermidine in supernatants of sulfide solid substrate conditions indicates that it can be used as a biomarker for sulfooxidizer activity (Martínez and Parada 2011). In order to further understand these phenomena, we searched for spermidine and polyamine transporters in the *A. ferrooxidans* strain ATCC 23270 genome, however, no candidates were found. Nevertheless, we identified genes, described in other bacteria such as *E. coli* and *V. cholerae*, that are associated to spermidine and putrescine transport (*pot*D and *pot*F; Igarashi and Kashiwagi 1999). The transport system for these polyamines is similar to ABC systems (secretion system type I), which has been well described in the literature. After analysis and comparison of the nucleotide and amino acid sequences of these genes in *A. ferrooxidans* ATCC 23270 and Wenelen strains, we detected functional candidates that contain ATP binding motifs (data not shown). We believe this could be a new secretion and/or incorporation model for spermidine in *A. ferrooxidans* which has not yet been identified. This finding confirms that the presence of spermidine has a functional significance for the bacterium, where the regulation is related to specific growth stages and sulfur presence.

#### Glutathione pathways

Glutathione related pathways seem more active in sulfur than in iron and chalcopyrite conditions for *A. ferrooxidans* strain Wenelen and *A. thiooxidans* strain Licanantay (Supplementary Table ST4). This observation is supported by Rohwerder and Sand (2003), who show that elemental sulfur activation provides a catalytic role for reduced glutathione (GSH). GSH is only detected in *A. ferrooxidans* strain Wenelen in the early exponential phase (T1), practically in the same amount as oxidized glutathione (GSSG). GSH is undetectable for either T2 or T3 time points for both bacteria, but two of the three constitutive amino acids, glutamate and glycine, are present (cysteine is undetectable under this technique). GSSG, in contrast, shows a concentration increase throughout the growth phases. These observations should be taken with caution, as it is likely that reduced glutathione could be oxidized during the extraction and/or identification protocol. Further analyses are needed to corroborate these observations.

Also, glutathione appears in more significant quantities in *A. thiooxidans* strain Licanantay than in *A. ferrooxidans* strain Wenelen.

#### Amino acids and metabolites involved in energy processes

*Acidithiobacillus thiooxidans* strain Licanantay shows a differential amino acid metabolic profile in chalcopyrite and sulfur conditions (Supplementary Table ST4). It seems that the presence of chalcopyrite triggers differential protein expression, most likely associated to cell attachment, energy source usage or detoxification mechanisms. A similar phenomenon is observed when comparing iron and sulfur conditions in *A. ferrooxidans* strain Wenelen (data not shown).

Because of the detection of glutamate and aspartate in supernatants (Supplementary Table ST3), we examined if these were related to structural polymers outside the cell (i.e. poly-glutamate and poly-aspartate). However, these polymers were undetectable in these cases (data not shown).

There is an increased presence of metabolites involved in energy processes (NADP+, ADP, AMP, CDP and dTDP) in *A. thiooxidans* strain Licanantay. This is concordant with the fact that sulfur processing is more effective in *A. thiooxidans* than in *A. ferrooxidans*. In addition, dihydroxyacetone phosphate and sedoheptulose 7-phosphate, involved in biofilm formation pathways, are enhanced in *A. thiooxidans* strain Licanantay when compared to *A. ferrooxidans* strain Wenelen (Supplementary Table ST4).

## Conclusions

This study introduces the first metabolomic analysis of two extremophilic biomining bacteria: *A. ferrooxidans* strain Wenelen and *A. thiooxidans* strain Licanantay. Principal component analysis indicates that metabolic profiles were different under each condition and therefore, metabolic results within each experiment are correlated.

Together with previous metabolic in silico reconstruction, our results show that several processes such as biofilm formation, carbon and amino acid usage, energy related compounds and oxidative stress response appear to be of importance for the bioleaching bacteria under study.

Attached and free cells have different metabolic profiles. Some interesting information emerged from the detailed analysis: spermidine, glutathione, and certain amino acids are the most abundant metabolites detected by our method (polar metabolites), which suggests that they have a potentially important role in the physiology of biomining bacteria. We believe that there is a relation between spermidine secretion and its role in biofilm formation in sulfur conditions and that an alternative polyamine synthesis pathway is present in these biomining bacteria. Further, we propose the use of spermidine as a biomarker for sulfooxidizer activity. Glutathione abundance and glutamate over-production in sulfur conditions, support previous knowledge about their participation in sulfur oxidation. Amino acid profiles vary between different growth conditions, suggesting differential protein synthesis possibly related to cell attachment, energy source or cell detoxification. Sugars are abundant in *A. thiooxidans* strain Lincanantay growing in sulfur media, and are likely involved in biofilm formation.

Unfortunately, directed functional mutagenesis has not been achieved for this type of bacteria. This complicates the study of relevant metabolic pathways identified in this work such as the spermidine synthesis pathway. However, the detection of potential new intermediates for this metabolic pathway and additional genomic analysis could be useful for elucidating new functional activities and synthesis. In order to enhance knowledge of metabolic processes related to bioleaching microorganisms, studies should focus on improving metabolic extraction methods. Also, additional metabolomic studies of organisms present in bioleaching processes are necessary to understand the effect of changing conditions (i.e. energy source, pH, toxicity, etc.). These studies should be conducted using a variety of detection techniques in order to include polar and non-polar metabolites. Our present study is the first step in consolidating metabolic knowledge for these biomining microorganisms, which will contribute to their global understanding and future applications.

## Electronic supplementary material

Below is the link to the electronic supplementary material.

Supplementary material 1 (DOC 37 kb)

Supplementary material 2 (XLSX 41 kb)

Supplementary material 3 (XLSX 534 kb)

Supplementary material 4 (XLSX 38 kb)

Supplementary material 5 (XLSX 49 kb)

Supplementary material 6 (XLSX 45 kb)

Supplementary material 7 (TIFF 145 kb)

Supplementary material 8 (TIFF 609 kb)

Supplementary material 9 (TIFF 528 kb)
