# Supplementary material for: Metabolomic study of Chilean biomining bacteria Acidithiobacillus ferrooxidans strain Wenelen and Acidithiobacillus thiooxidans strain Licanantay
Source: Metabolomics. 2012 Jul 21;9(1):247–57. doi: 10.1007/s11306-012-0443-3 (PMC3548112; doi:10.1007/s11306-012-0443-3)
Supplement: Supplementary file 1 — Supplementary material 1 (DOC 37 kb) [file 11306_2012_443_MOESM1_ESM.doc]

**Supplementary Material**

Supplementary Table ST1: 112 standards used in this study. Each is classified using a Kegg ID and detection mode. Peak detection was carried using the XCMS software. After detection, standard compounds were annotated based on a reference chromatogram. All peaks, both annotated and unknowns, were used in PCA analysis as variables differentiated by their m/z and migration time. Peak areas in each chromatogram correspond to the values used in PCA analysis.

Supplementary Table ST2: Migration time corrections for each chromatogram.

Supplementary Table ST3. Glutamate, Aspartate, and Spermidine concentrations (uM) in supernatants. For *A. ferrooxidans* strain Wenelen in iron and chalcopyrite conditions, there was no detection (ND). W: *A. ferrooxidans* strain Wenelen; L: *A. thiooxidans* strain Licanantay. Elemental sulfur (S^0^) or chalcopyrite (cpy) energy sources.

Supplementary Table ST4: Concentration (uM) of compounds in analyzed pathways according to organism and energy source.

Supplementary Table ST5: Pathway IDs used in Figures S1 and S2.

Supplementary Figure SF1: Cell concentrations for *A. ferrooxidans* strain Wenelen and *A. thiooxidans* strain Licanantay in different energy sources and times points T1, T2 y T3). All cultures had an initial concentration of 1.0E+06 [cell/mL].

Supplementary Figure SF2: Scored pathway for *A. ferrooxidans* strain Wenelen. Each pathway was scored based on the ratio of the 112 standards detected in each condition over the 112 standards that were reported to be in the pathway. Pathways lacking standards were removed. For the heatmap construction, each column was normalized. Red represents the highest value, yellow the midpoint and black the lowest.

Supplementary Figure SF3: Scored pathway for *A. thiooxidans* strain Licanantay. Each pathway was scored based on the ratio of 112 standards detected in each condition over the 112 standards that were reported to be in the pathway. Pathways lacking standards were removed. For the heatmap construction, each column was normalized, red represents the highest value, yellow the midpoint and black the lowest.
