# Supplementary figures and images for: Metabolomic study of Chilean biomining bacteria Acidithiobacillus ferrooxidans strain Wenelen and Acidithiobacillus thiooxidans strain Licanantay
Source: Metabolomics. 2012 Jul 21;9(1):247–57. doi: 10.1007/s11306-012-0443-3 (PMC3548112; doi:10.1007/s11306-012-0443-3)

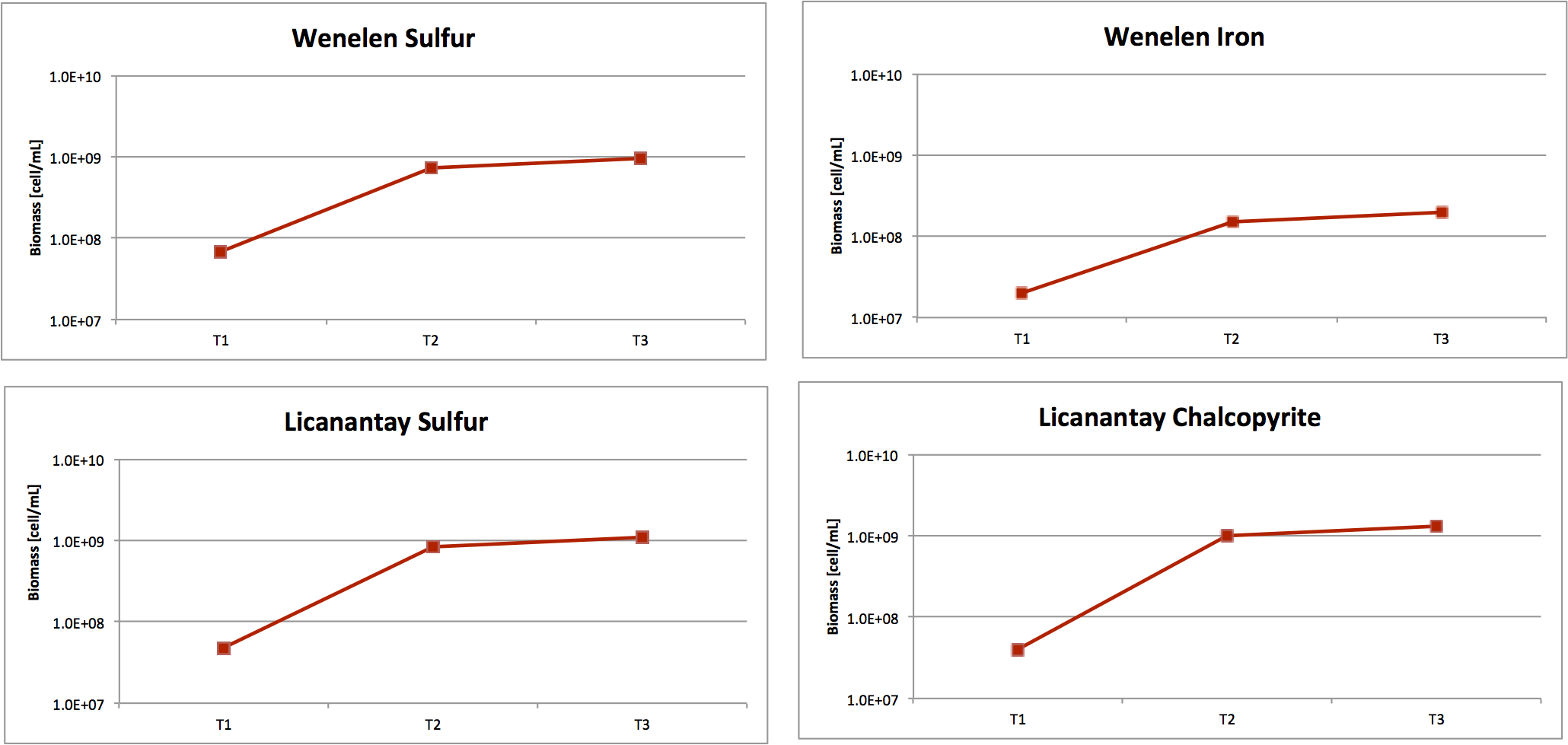

Supplement: Supplementary file 7 — Supplementary material 7 (TIFF 145 kb) [file 11306_2012_443_MOESM7_ESM.tiff]

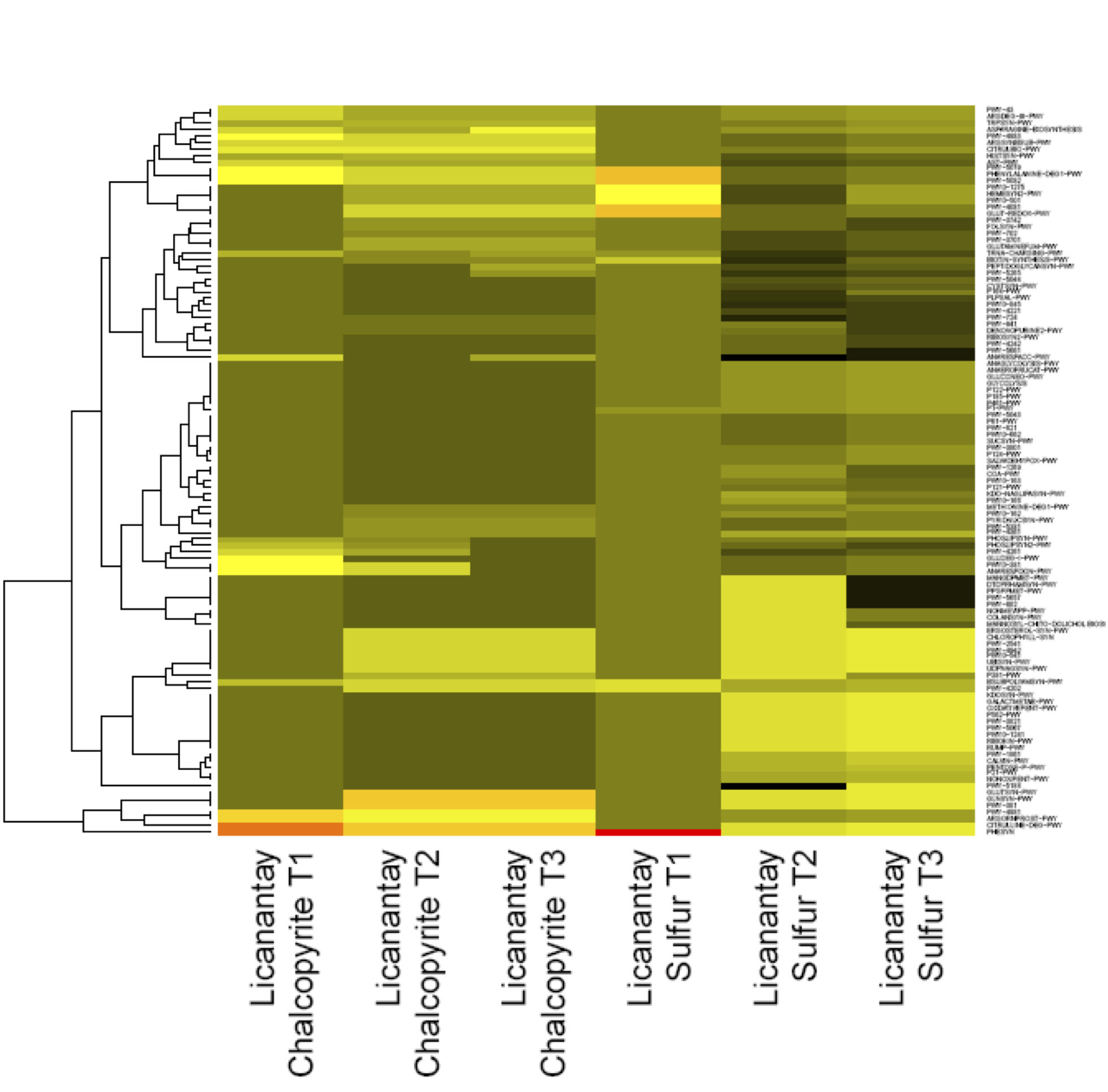

Supplement: Supplementary file 8 — Supplementary material 8 (TIFF 609 kb) [file 11306_2012_443_MOESM8_ESM.tiff]

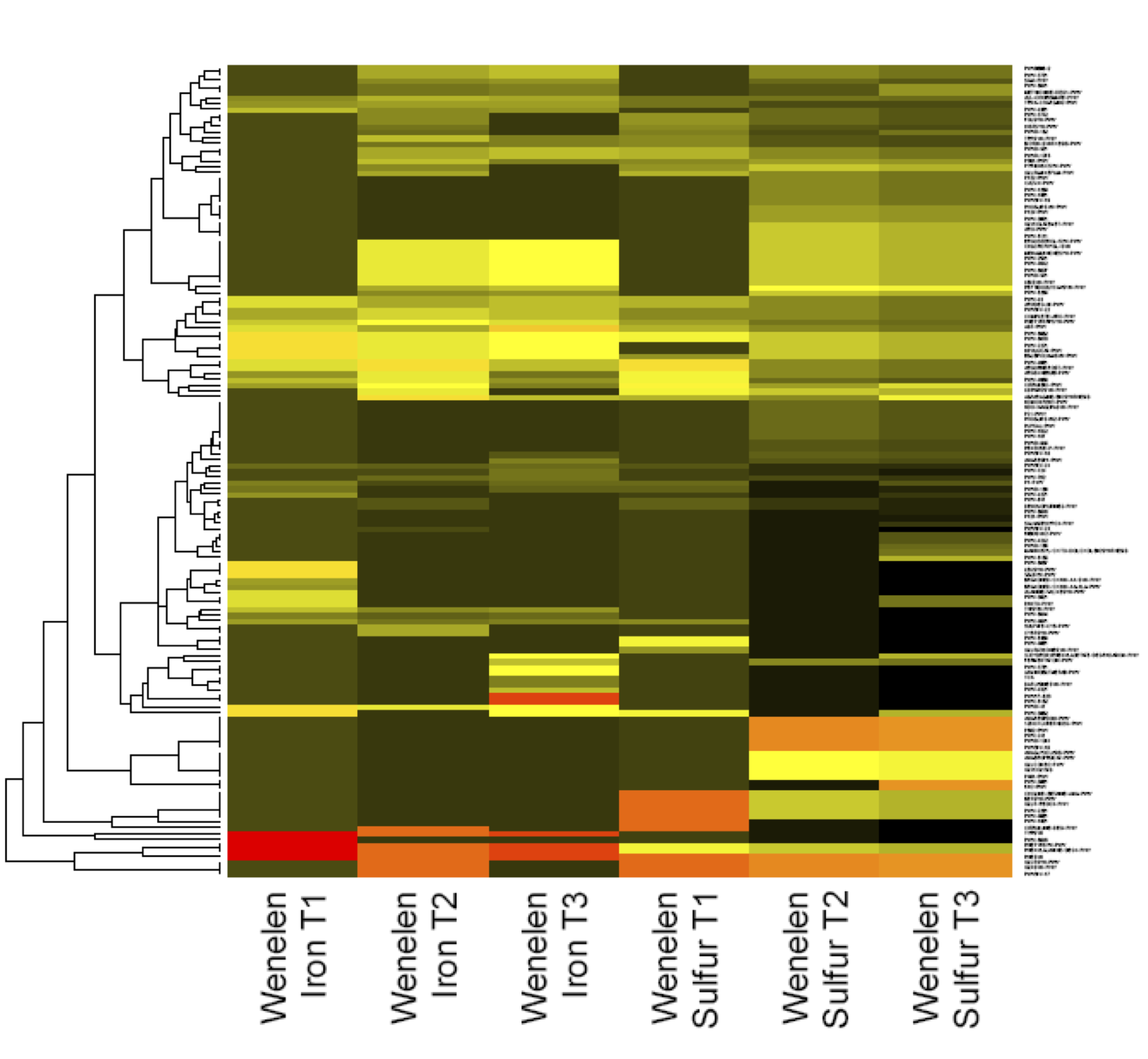

Supplement: Supplementary file 9 — Supplementary material 9 (TIFF 528 kb) [file 11306_2012_443_MOESM9_ESM.tiff]
